# Supplementary material for: Iterative improvement in the automatic modular design of robot swarms
Source: PeerJ Comput Sci. 2020 Dec 7;6:e322. doi: 10.7717/peerj-cs.322 (PMC7924708; doi:10.7717/peerj-cs.322)
Supplement: Supplemental Information 3 [file peerj-cs-06-322-s003.zip › argos3/doc/api/standalone/a00462_source.html]

ARGoS: plugins/robots/foot-bot/simulator/footbot\_distance\_scanner\_rotzonly\_sensor.cpp Source File


- Main Page
- Related Pages
- Namespaces
- Classes
- Files

- File List
- File Members

# plugins/robots/foot-bot/simulator/footbot\_distance\_scanner\_rotzonly\_sensor.cpp

Go to the documentation of this file.

```
00001 
00007 #include "footbot_distance_scanner_rotzonly_sensor.h"
00008 #include <argos3/core/simulator/entity/composable_entity.h>
00009 #include <argos3/core/simulator/entity/controllable_entity.h>
00010 #include <argos3/core/simulator/simulator.h>
00011 #include <argos3/core/simulator/space/space.h>
00012 
00013 namespace argos {
00014 
00015    /****************************************/
00016    /****************************************/
00017 
00018    static const Real FOOTBOT_RADIUS           = 0.085036758f;
00019 
00020    static const Real SHORT_RANGE_MIN_DISTANCE = 0.0f;
00021    static const Real SHORT_RANGE_RAY_START    = FOOTBOT_RADIUS;
00022    static const Real SHORT_RANGE_RAY_END      = FOOTBOT_RADIUS + 0.26f;
00023 
00024    static const Real LONG_RANGE_MIN_DISTANCE  = 0.12f;
00025    static const Real LONG_RANGE_RAY_START     = FOOTBOT_RADIUS;
00026    static const Real LONG_RANGE_RAY_END       = FOOTBOT_RADIUS + 1.42f;
00027 
00028    static const Real SENSOR_ELEVATION         = 0.123199866f;
00029 
00030    /****************************************/
00031    /****************************************/
00032 
00033    CFootBotDistanceScannerRotZOnlySensor::CFootBotDistanceScannerRotZOnlySensor() :
00034       m_pcRNG(NULL),
00035       m_bAddNoise(false),
00036       m_cSpace(CSimulator::GetInstance().GetSpace()),
00037       m_bShowRays(false) {}
00038 
00039    /****************************************/
00040    /****************************************/
00041 
00042    void CFootBotDistanceScannerRotZOnlySensor::Init(TConfigurationNode& t_tree) {
00043       try {
00044          CCI_FootBotDistanceScannerSensor::Init(t_tree);
00045          /* Show rays? */
00046          GetNodeAttributeOrDefault(t_tree, "show_rays", m_bShowRays, m_bShowRays);
00047          /* Noise range */
00048          GetNodeAttributeOrDefault(t_tree, "noise_range", m_cNoiseRange, m_cNoiseRange);
00049          if(m_cNoiseRange.GetSpan() > 0.0f) {
00050             m_bAddNoise = true;
00051             m_pcRNG = CRandom::CreateRNG("argos");
00052          }
00053       }
00054       catch(CARGoSException& ex) {
00055          THROW_ARGOSEXCEPTION_NESTED("Initialization error in foot-bot distance scanner rot_z_only sensor.", ex);
00056       }
00057    }
00058 
00059    /****************************************/
00060    /****************************************/
00061 
00062    void CFootBotDistanceScannerRotZOnlySensor::SetRobot(CComposableEntity& c_entity) {
00063       m_pcEmbodiedEntity = &(c_entity.GetComponent<CEmbodiedEntity>("body"));
00064       m_pcControllableEntity = &(c_entity.GetComponent<CControllableEntity>("controller"));
00065       m_pcDistScanEntity = &(c_entity.GetComponent<CFootBotDistanceScannerEquippedEntity>("distance_scanner"));
00066       m_pcDistScanEntity->Enable();
00067    }
00068 
00069    /****************************************/
00070    /****************************************/
00071 
00072    void CFootBotDistanceScannerRotZOnlySensor::Update() {
00073       /* Clear the maps */
00074       m_tReadingsMap.clear();
00075       m_tShortReadingsMap.clear();
00076       m_tLongReadingsMap.clear();
00077       /* Perform calculations only if the sensor is on */
00078       if(m_pcDistScanEntity->GetMode() != CFootBotDistanceScannerEquippedEntity::MODE_OFF) {
00079          /* Update the readings wrt to device mode */
00080          if(m_pcDistScanEntity->GetMode() == CFootBotDistanceScannerEquippedEntity::MODE_POSITION_CONTROL) {
00081             /* Sensor blocked in a position */
00082             /* Recalculate the rays */
00083             CalculateRaysNotRotating();
00084             /* Save the rotation for next time */
00085             m_cLastDistScanRotation = m_pcDistScanEntity->GetRotation();
00086             /* Update the values */
00087             UpdateNotRotating();
00088          }
00089          else {
00090             /* Rotating sensor */
00091             /* Recalculate the rays */
00092             CalculateRaysRotating();
00093             /* Update the values */
00094             UpdateRotating();
00095             /* Save the rotation for next time */
00096             m_cLastDistScanRotation = m_pcDistScanEntity->GetRotation();
00097          }
00098       }
00099    }
00100 
00101    /****************************************/
00102    /****************************************/
00103 
00104    void CFootBotDistanceScannerRotZOnlySensor::Reset() {
00105       /* Clear the maps */
00106       m_tReadingsMap.clear();
00107       m_tShortReadingsMap.clear();
00108       m_tLongReadingsMap.clear();
00109       /* Zero the last rotation */
00110       m_cLastDistScanRotation = CRadians::ZERO;
00111    }
00112 
00113    /****************************************/
00114    /****************************************/
00115 
00116    void CFootBotDistanceScannerRotZOnlySensor::UpdateNotRotating() {
00117       /* Short range [0] */
00118       CRadians cAngle = m_cLastDistScanRotation;
00119       cAngle.SignedNormalize();
00120       Real fReading = CalculateReadingForRay(m_cShortRangeRays0[0], SHORT_RANGE_MIN_DISTANCE);
00121       m_tShortReadingsMap[cAngle] = fReading;
00122       m_tReadingsMap[cAngle] = fReading;
00123       /* Long range [1] */
00124       cAngle += CRadians::PI_OVER_TWO;
00125       cAngle.SignedNormalize();
00126       fReading = CalculateReadingForRay(m_cLongRangeRays1[0], LONG_RANGE_MIN_DISTANCE);
00127       m_tLongReadingsMap[cAngle] = fReading;
00128       m_tReadingsMap[cAngle] = fReading;
00129       /* Short range [2] */
00130       cAngle += CRadians::PI_OVER_TWO;
00131       cAngle.SignedNormalize();
00132       fReading = CalculateReadingForRay(m_cShortRangeRays2[0], SHORT_RANGE_MIN_DISTANCE);
00133       m_tShortReadingsMap[cAngle] = fReading;
00134       m_tReadingsMap[cAngle] = fReading;
00135       /* Long range [3] */
00136       cAngle += CRadians::PI_OVER_TWO;
00137       cAngle.SignedNormalize();
00138       fReading = CalculateReadingForRay(m_cLongRangeRays3[0], LONG_RANGE_MIN_DISTANCE);
00139       m_tLongReadingsMap[cAngle] = fReading;
00140       m_tReadingsMap[cAngle] = fReading;
00141    }
00142 
00143    /****************************************/
00144    /****************************************/
00145 
00146 #define ADD_READING(RAYS,MAP,INDEX,MINDIST)                 \
00147    cAngle += cInterSensorSpan;                              \
00148    cAngle.SignedNormalize();                                \
00149    fReading = CalculateReadingForRay(RAYS[INDEX],MINDIST);  \
00150    MAP[cAngle] = fReading;                                  \
00151    m_tReadingsMap[cAngle] = fReading;
00152 
00153 #define ADD_READINGS(RAYS,MAP,MINDIST)          \
00154    ADD_READING(RAYS,MAP,1,MINDIST)              \
00155    ADD_READING(RAYS,MAP,2,MINDIST)              \
00156    ADD_READING(RAYS,MAP,3,MINDIST)              \
00157    ADD_READING(RAYS,MAP,4,MINDIST)              \
00158    ADD_READING(RAYS,MAP,5,MINDIST)
00159 
00160    void CFootBotDistanceScannerRotZOnlySensor::UpdateRotating() {
00161       CRadians cInterSensorSpan = (m_pcDistScanEntity->GetRotation() - m_cLastDistScanRotation).UnsignedNormalize() / 6.0f;
00162       CRadians cStartAngle = m_cLastDistScanRotation;
00163       /* Short range [0] */
00164       CRadians cAngle = cStartAngle;
00165       cAngle.SignedNormalize();
00166       Real fReading = CalculateReadingForRay(m_cShortRangeRays0[0], SHORT_RANGE_MIN_DISTANCE);
00167       m_tShortReadingsMap[cAngle] = fReading;
00168       m_tReadingsMap[cAngle] = fReading;
00169       ADD_READINGS(m_cShortRangeRays0, m_tShortReadingsMap, SHORT_RANGE_MIN_DISTANCE);
00170       /* Short range [2] */
00171       cAngle = cStartAngle + CRadians::PI;
00172       cAngle.SignedNormalize();
00173       fReading = CalculateReadingForRay(m_cShortRangeRays2[0], SHORT_RANGE_MIN_DISTANCE);
00174       m_tShortReadingsMap[cAngle] = fReading;
00175       m_tReadingsMap[cAngle] = fReading;
00176       ADD_READINGS(m_cShortRangeRays2, m_tShortReadingsMap, SHORT_RANGE_MIN_DISTANCE);
00177       /* Long range [1] */
00178       cAngle = cStartAngle + CRadians::PI_OVER_TWO;
00179       cAngle.SignedNormalize();
00180       fReading = CalculateReadingForRay(m_cLongRangeRays1[0], LONG_RANGE_MIN_DISTANCE);
00181       m_tLongReadingsMap[cAngle] = fReading;
00182       m_tReadingsMap[cAngle] = fReading;
00183       ADD_READINGS(m_cLongRangeRays1, m_tLongReadingsMap, LONG_RANGE_MIN_DISTANCE);
00184       /* Long range [3] */
00185       cAngle = cStartAngle + CRadians::PI_OVER_TWO + CRadians::PI;
00186       cAngle.SignedNormalize();
00187       fReading = CalculateReadingForRay(m_cLongRangeRays3[0], LONG_RANGE_MIN_DISTANCE);
00188       m_tLongReadingsMap[cAngle] = fReading;
00189       m_tReadingsMap[cAngle] = fReading;
00190       ADD_READINGS(m_cLongRangeRays3, m_tLongReadingsMap, LONG_RANGE_MIN_DISTANCE);
00191    }
00192 
00193    /****************************************/
00194    /****************************************/
00195 
00196    Real CFootBotDistanceScannerRotZOnlySensor::CalculateReadingForRay(const CRay3& c_ray,
00197                                                                       Real f_min_distance) {
00198       /* Get the closest intersection */
00199       SEmbodiedEntityIntersectionItem sIntersection;
00200       if(GetClosestEmbodiedEntityIntersectedByRay(sIntersection,
00201                                                   c_ray,
00202                                                   *m_pcEmbodiedEntity)) {
00203          if(m_bShowRays) m_pcControllableEntity->AddIntersectionPoint(c_ray, sIntersection.TOnRay);
00204          /* There is an intersection! */
00205          Real fDistance = c_ray.GetDistance(sIntersection.TOnRay);
00206          if(fDistance > f_min_distance) {
00207             /* The distance is returned in meters, but the reading must be in cm */
00208             if(m_bShowRays) m_pcControllableEntity->AddCheckedRay(true, c_ray);
00209             return fDistance * 100.0f;
00210          }
00211          else {
00212             /* The detected intersection was too close */
00213             if(m_bShowRays) m_pcControllableEntity->AddCheckedRay(true, c_ray);
00214             return -1.0f;
00215          }
00216       }
00217       else {
00218          /* No intersection */
00219          if(m_bShowRays) m_pcControllableEntity->AddCheckedRay(false, c_ray);
00220          return -2.0f;
00221       }
00222    }
00223 
00224    /****************************************/
00225    /****************************************/
00226 
00227 /* Highly reuse the vectors to speed up the computation */
00228 #define CALCULATE_SHORT_RANGE_RAY(ANGLE,INDEX)              \
00229    m_cDirection.RotateZ(ANGLE);                             \
00230    m_cOriginRayStart = m_cDirection;                        \
00231    m_cOriginRayEnd = m_cDirection;                          \
00232    m_cOriginRayStart *= SHORT_RANGE_RAY_START;              \
00233    m_cOriginRayEnd *= SHORT_RANGE_RAY_END;                  \
00234    m_cRayStart = m_pcEmbodiedEntity->GetOriginAnchor().Position;  \
00235    m_cRayStart += m_cOriginRayStart;                        \
00236    m_cRayStart.SetZ(m_cRayStart.GetZ() + SENSOR_ELEVATION); \
00237    m_cRayEnd = m_pcEmbodiedEntity->GetOriginAnchor().Position;  \
00238    m_cRayEnd += m_cOriginRayEnd;                            \
00239    m_cRayEnd.SetZ(m_cRayEnd.GetZ() + SENSOR_ELEVATION);     \
00240    m_cShortRangeRays0[INDEX].Set(m_cRayStart, m_cRayEnd);   \
00241    m_cRayStart = m_pcEmbodiedEntity->GetOriginAnchor().Position;  \
00242    m_cRayStart -= m_cOriginRayStart;                        \
00243    m_cRayStart.SetZ(m_cRayStart.GetZ() + SENSOR_ELEVATION); \
00244    m_cRayEnd = m_pcEmbodiedEntity->GetOriginAnchor().Position;  \
00245    m_cRayEnd -= m_cOriginRayEnd;                            \
00246    m_cRayEnd.SetZ(m_cRayEnd.GetZ() + SENSOR_ELEVATION);     \
00247    m_cShortRangeRays2[INDEX].Set(m_cRayStart, m_cRayEnd);   \
00248                                                             \
00249 /* Highly reuse the vectors to speed up the computation */
00250 #define CALCULATE_LONG_RANGE_RAY(ANGLE,INDEX)               \
00251    m_cDirection.RotateZ(ANGLE);                             \
00252    m_cOriginRayStart = m_cDirection;                        \
00253    m_cOriginRayEnd = m_cDirection;                          \
00254    m_cOriginRayStart *= LONG_RANGE_RAY_START;               \
00255    m_cOriginRayEnd *= LONG_RANGE_RAY_END;                   \
00256    m_cRayStart = m_pcEmbodiedEntity->GetOriginAnchor().Position;  \
00257    m_cRayStart += m_cOriginRayStart;                        \
00258    m_cRayStart.SetZ(m_cRayStart.GetZ() + SENSOR_ELEVATION); \
00259    m_cRayEnd = m_pcEmbodiedEntity->GetOriginAnchor().Position;  \
00260    m_cRayEnd += m_cOriginRayEnd;                            \
00261    m_cRayEnd.SetZ(m_cRayEnd.GetZ() + SENSOR_ELEVATION);     \
00262    m_cLongRangeRays1[INDEX].Set(m_cRayStart, m_cRayEnd);    \
00263    m_cRayStart = m_pcEmbodiedEntity->GetOriginAnchor().Position;  \
00264    m_cRayStart -= m_cOriginRayStart;                        \
00265    m_cRayStart.SetZ(m_cRayStart.GetZ() + SENSOR_ELEVATION); \
00266    m_cRayEnd = m_pcEmbodiedEntity->GetOriginAnchor().Position;  \
00267    m_cRayEnd -= m_cOriginRayEnd;                            \
00268    m_cRayEnd.SetZ(m_cRayEnd.GetZ() + SENSOR_ELEVATION);     \
00269    m_cLongRangeRays3[INDEX].Set(m_cRayStart, m_cRayEnd);
00270 
00271    /****************************************/
00272    /****************************************/
00273 
00274    void CFootBotDistanceScannerRotZOnlySensor::CalculateRaysNotRotating() {
00275       /* We make the assumption that the foot-bot is rotated only around Z */
00276       /* Get the foot-bot orientation */
00277       CRadians cTmp1, cTmp2, cOrientationZ;
00278       m_pcEmbodiedEntity->GetOriginAnchor().Orientation.ToEulerAngles(cOrientationZ, cTmp1, cTmp2);
00279       /* Sum the distance scanner orientation */
00280       cOrientationZ += m_pcDistScanEntity->GetRotation();
00281       /* Calculate the 2D vector representing this rotation */
00282       CVector2 cAbsoluteOrientation(1.0, cOrientationZ);
00283       /* The short range sensors are oriented along the foot-bot local X */
00284       m_cDirection = CVector3::X;
00285       CALCULATE_SHORT_RANGE_RAY(cAbsoluteOrientation, 0);
00286       /* The short range sensors are oriented along the foot-bot local Y */
00287       m_cDirection = CVector3::Y;
00288       CALCULATE_LONG_RANGE_RAY(cAbsoluteOrientation, 0);
00289    }
00290 
00291    /****************************************/
00292    /****************************************/
00293 
00294    void CFootBotDistanceScannerRotZOnlySensor::CalculateRaysRotating() {
00295       /* We make the assumption that the foot-bot is rotated only around Z */
00296       /* Get the foot-bot orientation */
00297       CRadians cTmp1, cTmp2, cOrientationZ;
00298       m_pcEmbodiedEntity->GetOriginAnchor().Orientation.ToEulerAngles(cOrientationZ, cTmp1, cTmp2);
00299       /* Sum the distance scanner orientation */
00300       cOrientationZ += m_cLastDistScanRotation;
00301       /* Calculate the 2D vector representing this rotation */
00302       CVector2 cAbsoluteOrientation(1.0, cOrientationZ);
00303       /* The sensor is rotating, so calculate the span between each successive ray */
00304       CVector2 cInterSensorSpan(1.0f, (m_pcDistScanEntity->GetRotation() - m_cLastDistScanRotation).UnsignedNormalize() / 6.0f);
00305       /* The short range sensors are oriented along the foot-bot local X */
00306       m_cDirection = CVector3::X;
00307       CALCULATE_SHORT_RANGE_RAY(cAbsoluteOrientation, 0);
00308       CALCULATE_SHORT_RANGE_RAY(cInterSensorSpan, 1);
00309       CALCULATE_SHORT_RANGE_RAY(cInterSensorSpan, 2);
00310       CALCULATE_SHORT_RANGE_RAY(cInterSensorSpan, 3);
00311       CALCULATE_SHORT_RANGE_RAY(cInterSensorSpan, 4);
00312       CALCULATE_SHORT_RANGE_RAY(cInterSensorSpan, 5);
00313       /* The long range sensors are oriented along the foot-bot local Y */
00314       m_cDirection = CVector3::Y;
00315       CALCULATE_LONG_RANGE_RAY(cAbsoluteOrientation, 0);
00316       CALCULATE_LONG_RANGE_RAY(cInterSensorSpan, 1);
00317       CALCULATE_LONG_RANGE_RAY(cInterSensorSpan, 2);
00318       CALCULATE_LONG_RANGE_RAY(cInterSensorSpan, 3);
00319       CALCULATE_LONG_RANGE_RAY(cInterSensorSpan, 4);
00320       CALCULATE_LONG_RANGE_RAY(cInterSensorSpan, 5);
00321    }
00322 
00323    /****************************************/
00324    /****************************************/
00325 
00326    REGISTER_SENSOR(CFootBotDistanceScannerRotZOnlySensor,
00327                    "footbot_distance_scanner", "rot_z_only",
00328                    "Carlo Pinciroli [ilpincy@gmail.com]",
00329                    "1.0",
00330                    "The foot-bot distance scanner sensor (optimized for 2D).",
00331                    "This sensor accesses the foot-bot distance scanner sensor. For a complete\n"
00332                    "description of its usage, refer to the common interface.\n"
00333                    "In this implementation, the readings are calculated under the assumption that\n"
00334                    "the foot-bot is always parallel to the XY plane, i.e., it rotates only around\n"
00335                    "the Z axis. This implementation is faster than a 3D one and should be used\n"
00336                    "only when the assumption about the foot-bot rotation holds.\n\n"
00337                    "REQUIRED XML CONFIGURATION\n\n"
00338                    "  <controllers>\n"
00339                    "    ...\n"
00340                    "    <my_controller ...>\n"
00341                    "      ...\n"
00342                    "      <sensors>\n"
00343                    "        ...\n"
00344                    "        <footbot_distance_scanner implementation=\"rot_z_only\" />\n"
00345                    "        ...\n"
00346                    "      </sensors>\n"
00347                    "      ...\n"
00348                    "    </my_controller>\n"
00349                    "    ...\n"
00350                    "  </controllers>\n\n"
00351                    "OPTIONAL XML CONFIGURATION\n\n"
00352                    "It is possible to draw the rays shot by the distance scanner in the OpenGL\n"
00353                    "visualization. This can be useful for sensor debugging but also to understand\n"
00354                    "what's wrong in your controller. In OpenGL, the rays are drawn in cyan when\n"
00355                    "they are not obstructed and in purple when they are. In case a ray is\n"
00356                    "obstructed, a black dot is drawn where the intersection occurred.\n"
00357                    "To turn this functionality on, add the attribute 'show_rays=\"true\"' in the\n"
00358                    "XML as in this example:\n\n"
00359                    "  <controllers>\n"
00360                    "    ...\n"
00361                    "    <my_controller ...>\n"
00362                    "      ...\n"
00363                    "      <sensors>\n"
00364                    "        ...\n"
00365                    "        <footbot_distance_scanner implementation=\"rot_z_only\"\n"
00366                    "                                  show_rays=\"true\" />\n"
00367                    "        ...\n"
00368                    "      </sensors>\n"
00369                    "      ...\n"
00370                    "    </my_controller>\n"
00371                    "    ...\n"
00372                    "  </controllers>\n",
00373                    "Usable"
00374       );
00375 
00376 }
```

---

Generated on 10 Jul 2018 for ARGoS by 
 1.6.1
